# Paecilomycies japonica reduces repeated nicotine-induced neuronal and behavioral activation in rats

**DOI:** 10.1186/s12906-015-0739-8

**Published:** 2015-07-14

**Authors:** Minsook Ye, Hyunju Lee, Hyunsu Bae, Dae-Hyun Hahm, Hye-Jung Lee, Insop Shim

**Affiliations:** Department of Science in Korean Medicine, Graduate School, College of Korean Medicine, Kyung Hee University, 26 KyungHee-daero, Seoul, 130-701 South Korea; Department of Physiology, College of Korean Medicine, Kyung Hee University, Seoul, 130-701 Republic of Korea

**Keywords:** *Paecilomyces japonica (PJ*), Nicotine, Locomotor activity, Development sensitization, c-Fos, Tyrosine hydroxylase (TH), Dopamine

## Abstract

**Background:**

Many studies have demonstrated that repeated injections of nicotine can produce progressive increases in locomotor activity and enhanced expression of *c-fos* and tyrosine hydroxylase (TH) in brain dopaminergic areas. *Paecilomyces japonica* (*PJ*) is a herbal medicine that is commonly used to treat opiate and other addictions in Eastern Asia. However, its influence on nicotine addiction has not been examined. This study was carried out to investigate the effects of *PJ* on repeated nicotine-induced behavioral sensitization of locomotor activity and c-Fos and TH expression in the rat brain using immunohistochemistry.

**Methods:**

Rats were pretreated with *PJ* (10, 25, 50, 100, and 200 mg/kg, intraperitoneally) 30 min before repeated injections of nicotine (0.4 mg/kg, subcutaneously, twice daily for 7 days). Locomotor activity was measured in rats during 7-day nicotine treatments. On the seventh day, c-Fos and TH expression were assessed.

**Results:**

Pretreatment with *PJ* decreased the development of nicotine-induced sensitization, c-Fos expression in the nucleus accumbens and striatum, and TH expression in the ventral tegmental area. *PJ* decreased nicotine-induced locomotor activity by modulating brain dopaminergic systems.

**Conclusion:**

The results of the present study suggest that *PJ* may be a useful agent for preventing and treating nicotine addiction.

## Background

Cigarette smoking has been related to many life-menacing diseases including heart disease, cancer, and chronic obstructive pulmonary disease [1]. The World Health Organization reports that there are approximately 1.25 billion smokers worldwide at present and that global mortality because of tobacco-related diseases will likely increase from approximately five million deaths annually in 2004, to over ten million deaths per year in 2030 [2]. For these reasons, cigarette smoking is a massive health problem in the world. Despite the many methods that have been developed for smoking cessation [3, 4], abstaining from smoking is difficult because of the addictive nature of nicotine.

Nicotine is one of the major psychoactive ingredients in tobacco and it is the principal addictive component of tobacco smoke, which contributes to the harmful tobacco smoking habits [5]. Nicotine is a psychoactive drug affiliated with diverse neurobiological and behavioral effects. It is an agonist of nicotinic acetylcholine receptors [6]. Repeated injection of the psychomotor stimulant drugs such as nicotine produces behavioral sensitization, a phenomenon evidenced by a steady increase in nicotine-induced behavioral responses to repeated injections of nicotine [7]. The mechanisms of behavioral sensitization have been suggested as an animal model of drug addiction mainly associated with repeated administration of psychomotor stimulant drugs, and it has been implicated in the development of drug addiction [8] and drug-induced psychosis [9, 10].

The development of sensitization is defined as the transient sequence of cellular and molecular events brought on by psychomotor stimulant administration that causes lasting changes in neural function responsible for behavioral augmentation [11], and has been connected anatomically to the mesolimbic dopaminergic pathways [12]. Enhanced dopamine (DA) transmission in dopaminergic target areas, including the nucleus accumbens (NAc) and striatum, is thought to underlie the development of behavioral sensitization to psychomotor stimulant drugs [13]. The behavioral sensitization caused by chronic injections of psychomotor stimulants may be related to alterations of neurotransmission in the mesolimbic dopaminergic pathways [14]. The cellular proto-oncogene *c-fos* belongs to the immediate early gene family and its induction has been used as a marker for postsynaptic activation [15]. Many neurons react to excitations of sufficient magnitude and duration by expressing the proto-oncogene *c-fos* and immunocytochemical detection of the Fos protein has been used to map the neural circuitry acted on by a diversity of behavioral and pharmacological treatments [16]. Several studies have suggested that administration of nicotine causes changes of the gene expression c-Fos in dopaminergic terminal regions, such as the NAc, striatum, and prefrontal cortex [9].

Tyrosine hydroxylase (TH) is the rate-limiting enzyme of DA synthesis in the midbrain dopaminergic neurons of the ventral tegmental area (VTA) modulating locomotor activity and reward. TH has been used as a marker for biochemical changes induced by chronic psychomotor stimulant exposure [17]. The levels of TH expression in the VTA are regulated in response to the chronic administration of several addictive drugs [18]. In addition, several studies have shown that repeated administration of nicotine increases expression of TH and DA biosynthesis in the mesolimbic DA pathway [19, 20].

*Paecilomyces japonica* (*PJ*) is one of the cordyceps species and is artificially grown on silkworm larvae [21]. *PJ* has been applied for medicinal purposes owing to its extensive physiological activities [21]. In China, cordyceps has been used as a remedy for more than 300 years. Many studies have also reported that cordyceps includes antitumor activity [22], immune-modulating effects [22], antioxidant activity [23], anti-hyperglycemic activity [24], reproductive function enhancement activity [25], anti-fatigue activity [26], neurotrophic effects [27], and has a protective effect on the kidney and liver [28]. Traditionally, cordyceps has been used for centuries as a detoxification agent for opioid addiction [29]. It has been used both in the treatment of opioid addiction and for lead poisoning [30]. However, *PJ* has not yet been established to be effective for nicotine addiction by experimental methods, even though this is a likely effect of *PJ* considering the previous reports on therapeutic effects of *PJ* in other addictive drugs.

This study was conducted to investigate the effects of *PJ* on repeated nicotine-induced behavioral sensitization and neuronal activity. The efficacy of *PJ* for nicotine-induced behavioral and neural activation was investigated by assessing locomotor activity and by using immunohistochemical methods to determine a possible mechanism underlying the suppressive effects of *PJ* on repeated-nicotine-induced behavioral sensitization in rats.

## Methods

### Animals

The animals used in this study were 40 male Sprague–Dawley rats, weighing 250 to 270 g at the start of the experiment. Upon arrival, animals were randomly assigned to several groups and housed for at least eight days prior to experimental procedures. Rats were placed in a quiet, temperature- and humidity-controlled room (23 °C ± 2 °C and 60 % ± 5 %, respectively), and kept on a 12-h light, 12-h dark cycle in individual home cages with food and water available *ad libitum*. The current study was reviewed and approved by the Ethics Committee of the Kyung Hee University (KHUASP (SE)-13-041) and their care conformed to Guidelines of the US National Institutes of Health and Korean Academy of Medical Sciences

### Preparations of *Paecilomyces japonica* extract and drugs

*Paecilomyces japonica* (*PJ*) was purchased and identified from the Plant Extract Bank (H Max Pharmacy Co, Daejon, Korea). A voucher specimen of *PJ* has been deposited at the herbarium located at the College of Korean Medicine, Kyung Hee University (Reference No. KH-PJAM01). The dried *PJ* (200 g) was drenched in 10-fold volume aqueous-methanol (30:70) for 3 days on an ultrasound bath and filtered through a muslin cloth and Whatman filter paper (Maidstone, UK) concurrently. This process was repeated three times and all the pooled filterates were evaporated on a rotary evaporator (Eyela, Tokyo Rikakikai Co., Ltd., Japan), under reduced pressure (−760 mmHg) to get a dark greenish semi-solid material, yielding 20.7 wt/wt %.

### Animals and nicotine administration

Nicotine hydrogen tartrate was purchased from Sigma Chemical (St. Louis, MO, USA). Nicotine was dissolved in 0.9 % saline at a concentration of 0.5 mg mL^−1^, and was injected subcutaneously (s.c.) in a volume of 1 mL/1000 g.

Rats were randomly divided into seven groups. The development of nicotine addiction was produced by repeated injection of nicotine (0.4 mg/kg, s.c., free base equivalent) twice a day for 7 consecutive days. The vehicle-treated rats (as a negative control) were administered saline (0.9 % NaCl, s.c.) instead of nicotine in the same way (normal group). Another group was pretreated with *PJ* (10, 25, 50, 100, and 200 mg/kg, i.p. *PJ* group) intraperitoneally 30 min prior to the injection of saline in the development phase. The *PJ*-treated groups were divided as follows: 10 mg/kg *PJ* plus nicotine-treated group (*PJ*10 group), 25 mg/kg *PJ* plus nicotine-treated group (*PJ*25 group), 50 mg/kg *PJ* plus nicotine-treated group (*PJ*50 group), 100 mg/kg *PJ* plus nicotine-treated group (*PJ*100 group), and 200 mg/kg *PJ* plus nicotine-treated group (*PJ*200 group). The *PJ* treatments were performed i.p. 30 min prior to the injection of nicotine or saline.

### Measurement of locomotor activity

Locomotor activity was measured by a contrast-sensitive computer-controlled video tracking activity box in rats during 7 day nicotine treatments. The system was composed of a black ventilated test chamber (40 × 40 × 45), interior lighted with a ceiling-mounted video camera. The camera’s image was transmitted to a contrast-sensitive tracker that mapped the point of highest contrast and relayed the digitalized coordinates to a computer. Dedicated software stored the information and coincidently displayed a map of the tracked subject. Data were collected in 10 min time bins during 1 h after all drug injections.

### c-Fos and TH Immunohistochemistry

At the end of the locomotor session, animals were profoundly anesthetized with sodium pentobarbital (100 mg/kg, i.p), and then perfused transcardially with heparinized 0.9 % NaCl solution, followed by a 4 % solution of paraformaldehyde in 0.1 M phosphate-buffered saline (PBS; pH 7.4) for 10–15 min. The brains were removed and post-fixed in the same fixative for overnight. They were then placed in 20 % sucrose solution diluted in 0.1 M sodium phosphate buffer (PBS; pH 7.4) for 2 days at 4 °C. Brains were then frozen and coronal sections (30 μm thick) were cut on the NAc, striatum, and VTA using a cryostat (Leica CM1850; Leica Microsystems Ltd., Nussloch, Germany). The sections were obtained according to the rat atlas of Paxinos and Watson (80). The sections were immunostained for Fos and TH protein expression using the avidin-biotin-peroxidase method. Briefly, the sections were rinsed three times for 3 min each in PBS before being incubated in PBS containing 0.3 % Triton X-100 (PBST) containing 0.02 sodium azide, 2 % normal rabbit antibody or Fos primary antibody diluted 1:2000 (rabbit polyclonal antibody; Santa Cruz Biotechnology, Santa Cruz, CA, USA) and 2 % normal sheep antibody and TH primary antibody dilution 1:2000 (sheep polyclonal antibody; Chemicon International Inc., Temecular, CA, USA) for 72 h at 4 °C, respectively. After three more rinses in PBST, the sections were placed in Vectastain Elite ABC reagent (Vector Laboratories Ltd, Burlingame, CA, USA) for 2 h at room temperature. To visualize immunoreactivity, the sections were incubated for 90 min in avidin-biotin complex (ABC) reagent (Vectastain Elite ABC Kit; Vector Laboratories Ltd), washed three times for 5 min in PBS, and incubated in a solution containing 3,3'-diaminobenzidine (Sigma-Aldrich Chemical Co., St. Louis, MO, USA) and 0.01 % H_2_O_2_ for 1 min. Finally, the tissues were washed in PBS and incubations were carried out in an orbital shaker, followed by a brief rinse in distilled water, and individually mounted onto slides. Slides were allowed to air dry, and were then cover slipped. Images were captured using a DP2-BSW imaging system (Olympus, CA, USA) and processed using Adobe Photoshop (Adobe Systems, Inc., San Jose, CA, USA). The sections were viewed at 100× magnification and the number of cells within 200 × 200 μm grids was counted by an observer blinded to the experimental groups. The cells within the NAc, striatum, and VTA were counted in at least three different sections for each rat.

### Statistical analysis

Statistical comparisons were conducted for the behavioral and histochemical studies using one-way or repeated-measures analysis of variance, respectively, and Tukey’s *post hoc* tests were conducted. All of the results are presented as means ± S.E.M., and we used SPSS 15.0 for Windows for analysis of the statistics. The significance level was set at *P* < 0.05.

## Results

### Effects of *PJ* on Nicotine-Induced Behavioral Sensitization

**Fig. 1:**
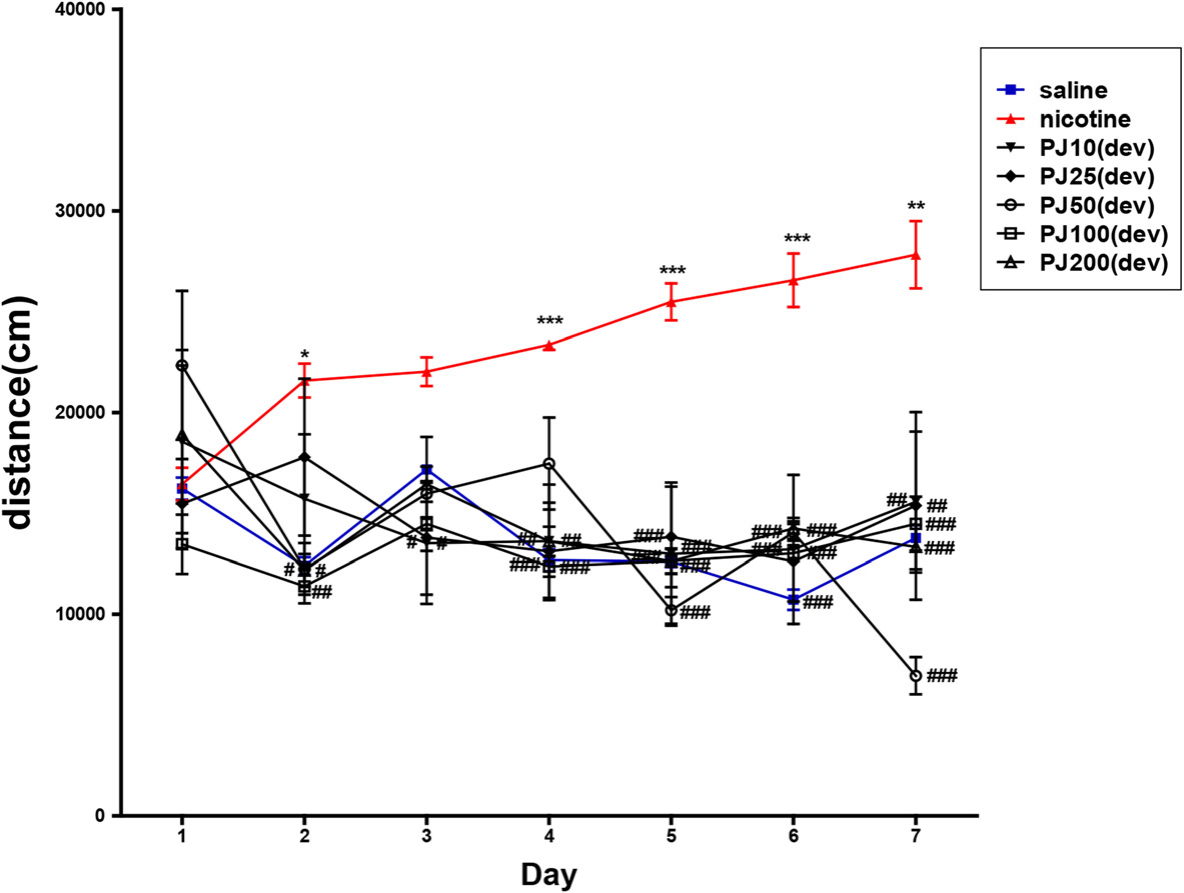
Effects of *PJ* on nicotine-induced locomotor sensitization. Rats were pretreated with vehicle (▲), *PJ* (10 mg/kg, i.p., ▼ *PJ*10(dev)), *PJ* (25 mg/kg, i.p., ◆ *PJ*25(dev)), *PJ* (50 mg/kg, i.p., ● *PJ5*0(dev)), *PJ* (100 mg/kg, i.p., □ *PJ*100(dev)), or *PJ* (200 mg/kg, i.p., △ *PJ20*0(dev)) 30 min before injections of nicotine twice daily for 7 consecutive days. The saline-treated group was treated with only saline (■). * = significant difference from saline-treated, nicotine-treated group and # from saline or *PJ*-pretreated and nicotine-treated group: *, *P* < 0.05; **, *P* < 0.01; ***, *P* < 0.001; #, *P* < 0.05; ##, *P* < 0.01; ###, *P* < 0.001. Vertical lines indicate S.E.M. (N = 6–7)

The effects of *PJ* on repeated nicotine-induced hyperactivity during the 7-day development phase are shown in Fig. 1. A repeated-measures analysis of variance (7 × 8, treatment × time) performed on the locomotor activity following the *PJ* treatments for the development test indicated a significant effect of drug [F(6,40) = 28.240, *p* < 0.001], a significant effect of time [F(7,42) = 3.964, *p* < 0.001], and a significant interaction between drug × time [F(42,280) = 3.481, *p* < 0.001]. The administration of *PJ* (10, 25, 50, and 100 mg/kg) before nicotine injection, during the 7 day development phase, significantly inhibited the development of nicotine-induced sensitization during the 7 day nicotine treatments (Fig. 1). Tukey’s post-hoc comparisons indicated that repeated injection of nicotine produced significant increases in locomotor activity, compared with those of saline-pretreated rats on day 7 (*p* < 0.001).

### Effects of *PJ* on Nicotine-induced Fos-like Immunoreactivity in the NAc and striatum

**Fig. 2:**
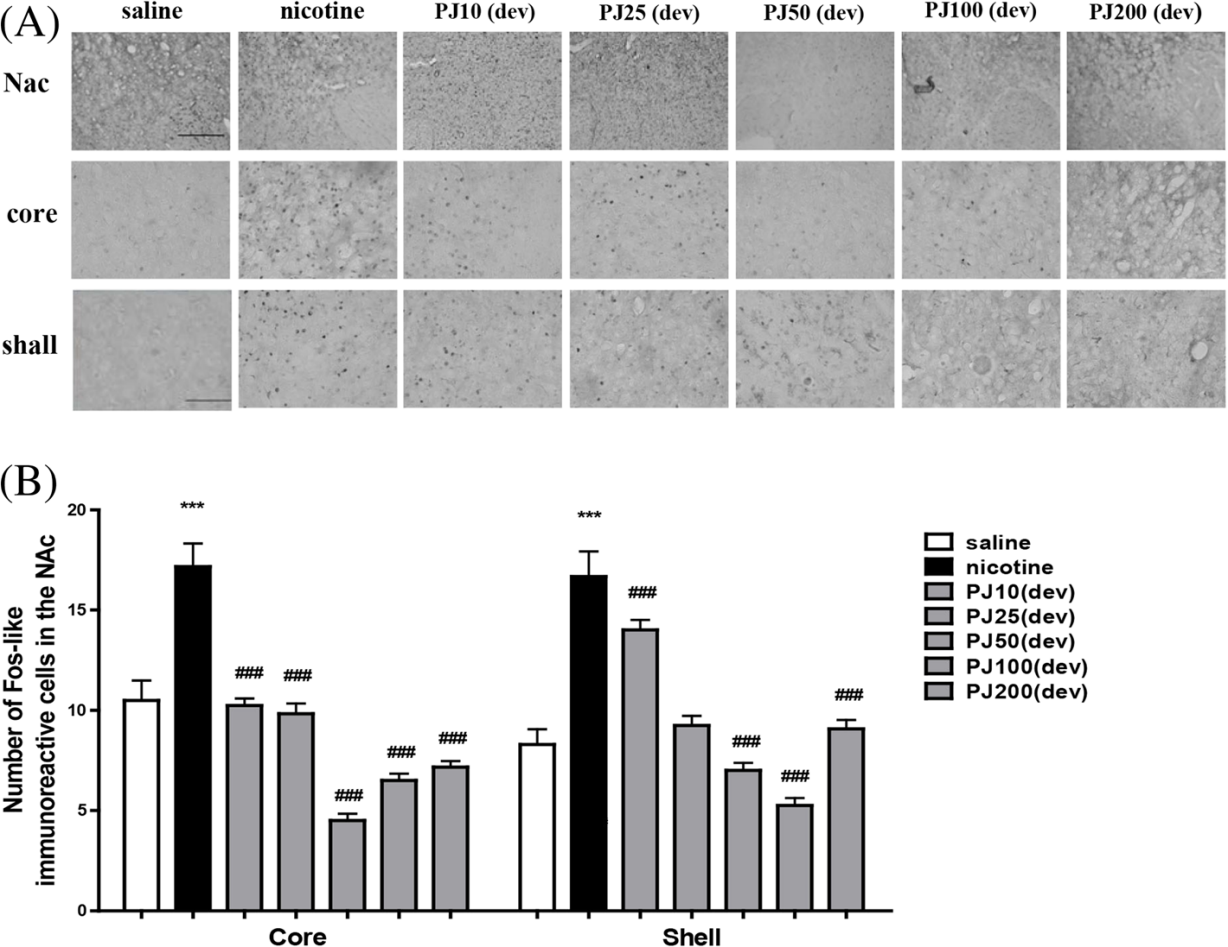
Expression of c-Fos in the NAc of rat brain after systemic injections of nicotine with *PJ*. Rats were pretreated with *PJ* (10, 25, 50, 100, 200 mg/kg) 30 min before nicotine injection (0.4 mg/kg). Expression of c-Fos in the NAc after systemic injections of saline, repeated nicotine or repeated nicotine with *PJ*. Representative images are displayed (**a**). The scale bars represent 100 μm and 200 μm. The number of c-Fos cells in the NAc (**b**). * Significant difference from saline-treated value (only shown for nicotine-treated) ***, *P* < 0.001, # Significant difference from nicotine-treated value ###, *P* < 0.001. a; saline, b; nicotine, c; *PJ*10 + nicotine, d; *PJ*25 + nicotine, e; *PJ*50 + nicotine, f; *PJ*100 + nicotine, g; *PJ*200 + nicotine

In rats receiving repeated nicotine, Fos-like immunoreactivity (FLI) was increased, in a variety of brain areas. Following the systemic injections of nicotine, a massive amount of FLI was present in the NAc [F (6,83) = 88.451, *p* < 0.001, Fig. 2]. In the NAc, post-hoc comparisons revealed that the nicotine-treated group showed a marked increase in the FLI compared with the saline-treated group (*P* < 0.001, Fig. 2). Pretreatment with *PJ* (10, 25, 50, 100, and 200 mg/kg) 30 min before the nicotine injection decreased the numbers of Fos-like immunoreactive cells to 24.25 ± 0.52 (*p* < 0.001), 19.08 ± 0.61 (*p* < 0.001), 11.5 ± 0.34 (*p* < 0.001), 11.75 ± 0.34 (*p* < 0.001), and 16.25 ± 0.53 (*p* < 0.001), respectively, as compared with Fos-like immunoreactive cells of 33.83 ± 1.71 in the nicotine-treated group (Fig. 2).

**Fig. 3:**
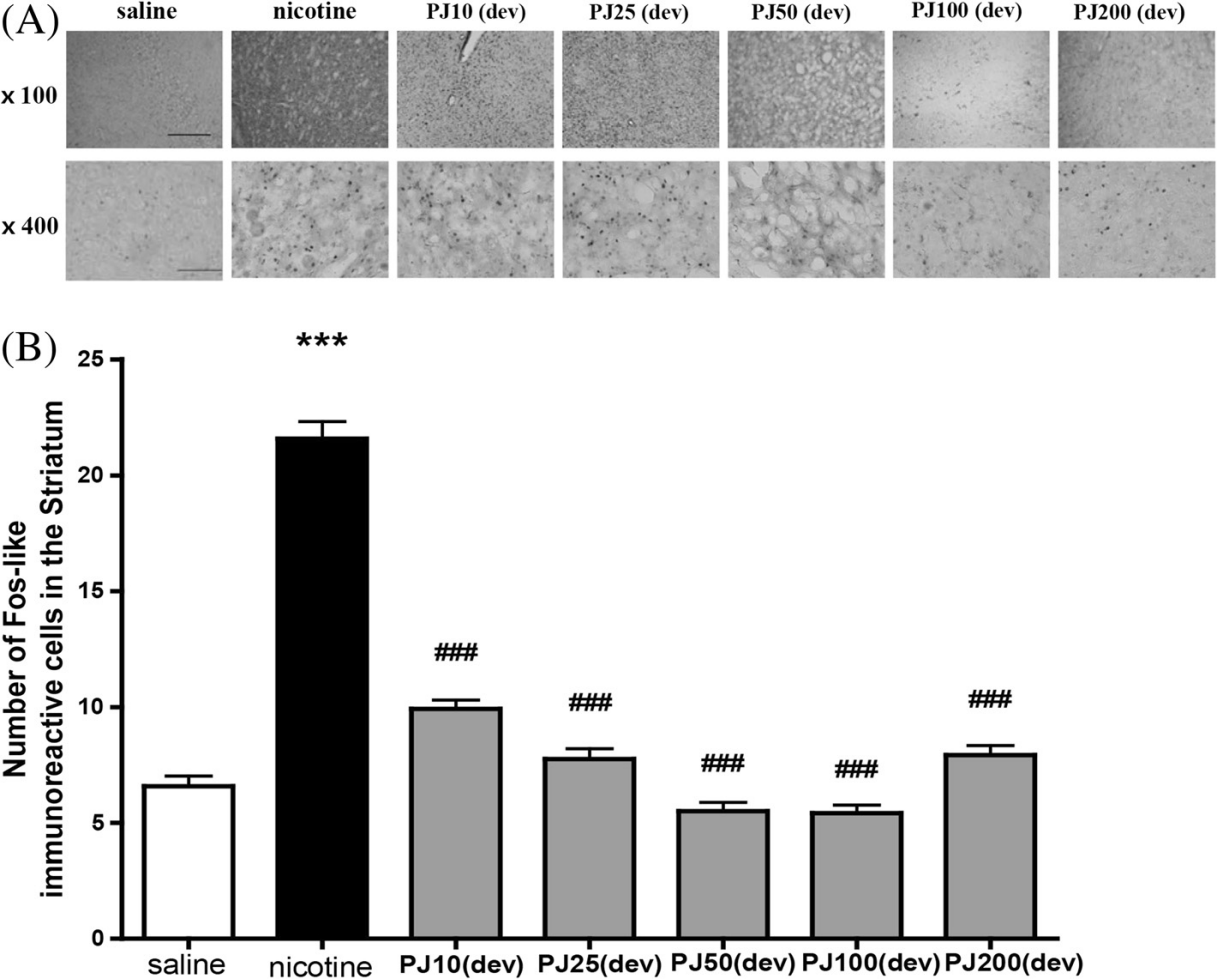
Expression of c-Fos in the striatum of rat brain after systemic injections of nicotine with *PJ*. Expression of c-Fos in the striatum after systemic injections of saline repeated nicotine with *PJ*. Representative images are displayed (**a**). The scale bars represent 100 μm and 200 μm. The number of c-Fos cells in the striatum measured (**b**). * Significant difference from saline-treated value (only shown for nicotine-treated) ***, *P* < 0.001, # Significant difference from nicotine-treated value ###, *P* < 0.001. a; saline, b; nicotine, c; *PJ*10 + nicotine, d; *PJ*25 + nicotine, e; *PJ*50 + nicotine, f; *PJ*100 + nicotine, g; *PJ*200 + nicotine

In the striatum, post-hoc comparisons revealed that the nicotine-treated group showed a marked increase in the FLI compared with the saline-treated group [F(6,83) = 39.737, *p* < 0.001, Fig. 3]. Pretreatment with *PJ* (10, 25, 50, 100, and 200 mg/kg) 30 min before the nicotine injection decreased the numbers of Fos-like immunoreactive cells to 9.92 ± 0.39 (*p* < 0.001), 7.75 ± 0.45 (*p* < 0.001), 5.5 ± 0.38 (*p* < 0.001), 5.41 ± 0.35 (*p* < 0.001), and 7.92 ± 0.42 (*p* < 0.001), respectively, as compared with Fos-like immunoreactive cells of 21.58 ± 0.74 in the nicotine-treated group (Fig. 3).

### Effects of *PJ* on Nicotine-induced TH-like Immunoreactivity in the VTA

**Fig. 4:**
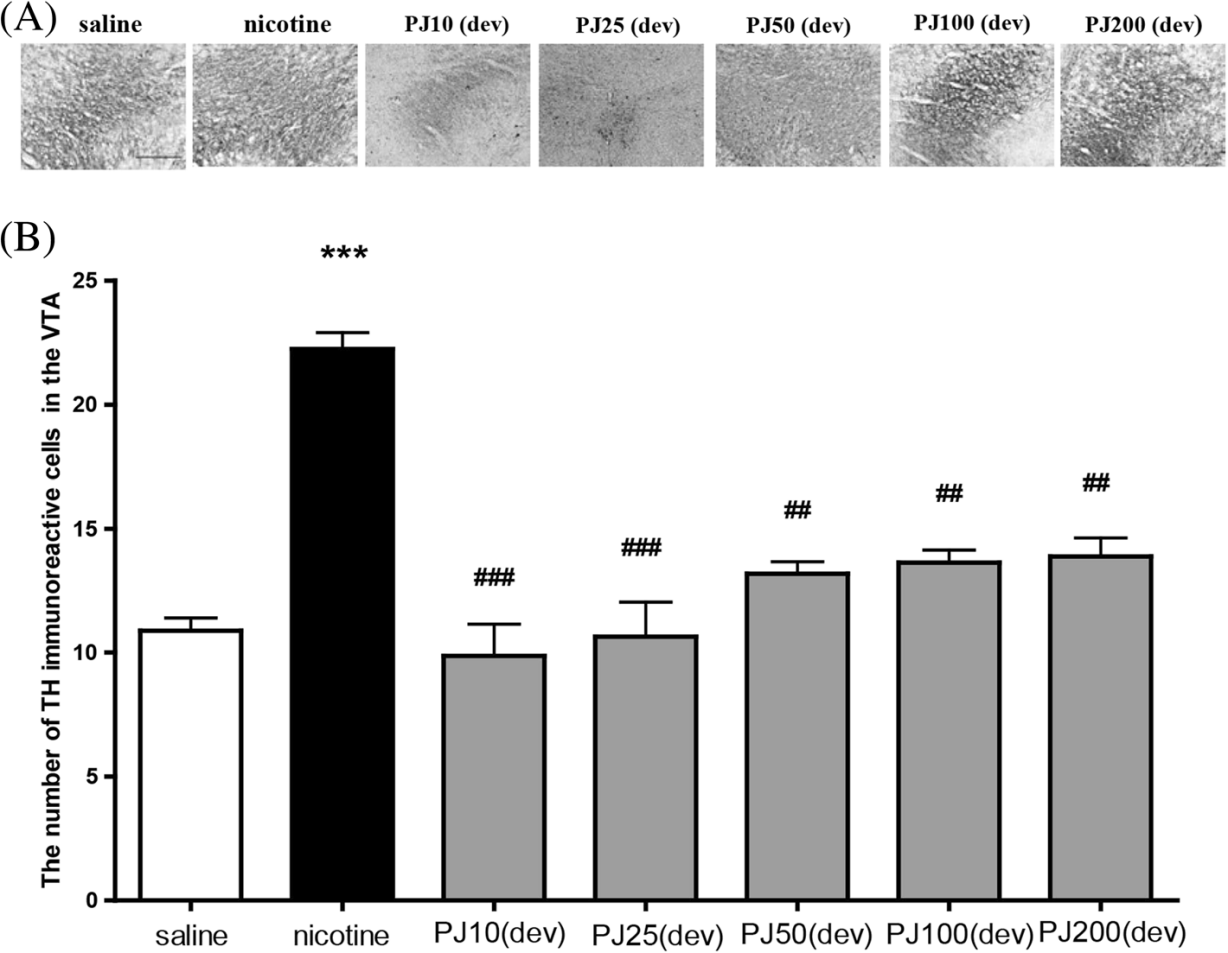
Expression of TH in the VTA of rat brain after systemic injections of nicotine with *PJ*. Expression of TH in the VTA after systemic injections of saline repeated nicotine or repeated nicotine with *PJ*. Representative images are displayed (**a**). The scale bars represent 100 μm. The number of TH-positive neurons in the VTA (**b**). * Significant difference from saline-treated value (only shown for nicotine-treated) ***, *P* < 0.001, # Significant difference from nicotine-treated value ##, *P* < 0.01; ###, *P* < 0.001. a; saline, b; nicotine, c; *PJ*10 + nicotine, d; *PJ*25 + nicotine, e; *PJ*50 + nicotine, f; *PJ*100 + nicotine, g; *PJ*200 + nicotine

Following the systemic injection of nicotine, a massive amount of TH-like immunoreactivity was present in the VTA of the nicotine-treated group, compared with the saline-treated group. Nicotine-treated group immunoreactivities were highly enriched in the VTA [F(6,83) = 15.598, *p* < 0.001, Fig. 4]. This effect was significantly lessened by *PJ* treatment during the development of sensitization. The mean number of neurons showing TH-like immunoreactivity in examined regions of the VTA was 21.58 ± 3.61 and 19.61 ± 2.72, for rats given repeated treatment. However, a much smaller number of immunoreactive cells could be seen in these areas of the *PJ*-treated group. The post-hoc comparisons revealed that the nicotine-treated group showed a greater increase in TH expression than the saline-treated group (*p* < 0.01). The administrations of 10, 25, 50, 100, and 200 mg/kg-body weight *PJ*, 30 min before the nicotine injection, decreased the numbers of TH-like immunoreactive cells to 27.39 ± 1.10 (*p* < 0.001), 22.83 ± 0.76 (*p* < 0.001), and 21.67 ± 0.75 (*p* < 0.001), respectively, as compared with TH-like immunoreactive cells of 29.56 ± 2.32 in the nicotine-treated group.

## Discussion

In the present study, the effects of *PJ* on nicotine-induced behavioral sensitization were compared, examined, and related to modulation of the activation of dopaminergic neurons. The results show that pretreatment with *PJ* during the development phase blocked the induction of behavioral sensitization and expression of c-Fos and TH. *PJ* is a codyceps species that is a well-known traditional medicine. Our results suggest that *PJ* can block the sensitization produced by repeated injections of addictive drugs. *PJ* may be useful in treating or preventing addiction-related behaviors and neural activity.

The results of this study demonstrate that repeated daily administrations of nicotine produced a large increase in locomotor activity. The present study also demonstrated increased Fos-like immunoreactivity in the NAc and striatum and TH-like immnoreactivity in the VTA, which are the major projection areas of the dopaminergic system. These results are concordant with previous findings [31]. The development of sensitization to psychomotor stimulants leads to enduring changes in neural function responsible for behavioral augmentation. A variety of data sets show that these processes are functionally and anatomically evident [11]. Several studies suggest that different brain areas and different mechanisms are associated with the development of sensitization to psychostimulant drugs such as nicotine [12]. Development of behavioral sensitization to psychostimulants occurs in the VTA [32, 33], which is the locus of the dopamine cells that lead to the mesocorticolimbic dopamine pathway [34]. Several studies have shown that the VTA is related to the development phase of drug-induced sensitization [14]. For example, intra-VTA injections of SCH 23390 blocked both the cocaine-induced increase in extracellular dopamine in the NAc and the development of sensitized responses [35]. Furthermore, antagonists of dopamine D1 and glutamate N-methyl-D-aspartate receptors or agonists of GABA_B_ receptors administered locally in the VTA can arrest the development of amphetamine sensitization [36]. These results suggest that the VTA is related to the development of sensitization and the effects of *PJ* during this development. The neural mechanisms by which *PJ* is involved in nicotine-induced development of sensitization remain to be fully elucidated.

Increased TH expression and dopamine release are important factors in nicotine addiction [37]. Our findings regarding expression of TH protein in the VTA indicate a fine agreement with previous findings that injection of nicotine elevates the extracellular DA levels and its metabolites in the VTA [20, 38]. These data suggest that pretreatment with *PJ* significantly suppressed the nicotine-induced TH expression in the VTA, suggesting that the suppressive effects of *PJ* are related to blockade of dopaminergic biosynthesis or transmission.

Numerous studies have used c-fos expression mapping approaches for the identification of neural circuits underlying behavioral sensitization to abused drugs [39]. The expression of c-Fos increases significantly in the reward circuit-related areas after acute treatment with nicotine [40]. Previous studies have demonstrated that chronic administration of nicotine induced the expression of c-Fos in dopaminergic target areas, such as the NAc and striatum [41], which are considered an important region in the mediation of anticipatory and reward responses [42]. The induction of c-Fos protein in the NAc and striatum indicated a fine agreement with the previous findings that repeated injection of nicotine elevates the extracellular DA levels and its metabolites in the NAc and striatum [20]. In the present study, effects of *PJ* on nicotine-induced Fos-like immunoreactivity in the NAc and striatum were compared with saline across the development phase. We show that pretreatment with *PJ* inhibits Fos-like immunoreactivity in the NAc and striatum during the development of sensitization. These results suggest that pretreatment with *PJ* may attenuate Fos-like immunoreactivity by modulating the activities of postsynaptic dopamine receptors in the NAc and striatum.

Cordyceps has a long history for use in drug abuse therapy. Its therapeutic efficacy has been confirmed by many medical studies in the Compendium of Materia Medica (a compendium of Chinese Traditional medicines complied by Li Shizhen). According to a recent study, cordyceps are reported to have pro-recovery effects in severe drug addicts as reported in a clinical trial in Switzerland. The mechanism of *PJ* in drug abuse therapy has not been investigated. In most cases, cordyceps are a type of traditional Chinese tonic that has already shown a positive effect on some human body systems. Cordyceps have many pharmacological ingredients, such as cordycepin, polysaccharides, ergosterol and its analogs, mannitol, and peptides [43]. A recent study has reported that mannitol affects nicotine-induced neurotransmitter release [44]. Cordyceps have several bioactivities, such as aphrodisiac [45, 46], analgesic [47], immune modulator [48], antioxidant [49], antitumor [50], and antibacterial [51], and also have protective effects on the kidney [50] and liver [28]. Furthermore, cordyceps have neurotrophic effects [27], which have been found to modulate several addictive behaviors related to the actions of certain drugs of abuse [52]. Several studies show that neurotrophic factors and their signaling pathways play an important role in mediating drug-induced changes in brain reward systems and behavioral sensitization [53]. Consequently, pharmacological compounds in cordyceps may be associated with neurotrophic effects, which regulate behavioral sensitization and reward circuits of drug addiction. Despite this suggestion, the effects of *PJ* concerning bioactivities and action mechanisms have not yet been clearly analyzed. Additionally, in neither study were there any reports of drug addiction related to *PJ*.

The present study showed that pretreatment with *PJ* during the development phase also blocked the increased locomotor activity to subsequent nicotine challenge. These results are in good agreement with previous studies showing that pretreatment with MK-801 and clozapine blocked the development of sensitization to drugs of abuse such as nicotine [54]. It is likely that the observed blockade of *PJ* on behavioral activity is closely associated with the blockade of dopaminergic biosynthesis or transmission, shown by reduced post-synaptic neuronal activation in dopaminergic terminals, the Nac and striatum and pre-synaptic neuronal activation in the VTA. These results suggest that *PJ* could be effective for ameliorating the behavioral responses of nicotine dependence, possibly by modulating the central dopaminergic system, and it may be a useful therapeutic target for developing a novel drug for treating nicotine addiction. However, more direct measures of behavioral sensitization, such as nicotine self-administration or nicotine-induced conditioned place preference, should be tested in the near future, to determine the inhibitory effects of *PJ* on nicotine-dependent behavioral patterns.

## Conclusions

*Paecilomyces japonica* decreased the development of nicotine-induced behavioral sensitization and c-Fos and TH expression in the brain. *PJ* decreased the locomotor activity of nicotine by possibly modulating the central dopaminergic system, suggesting that *PJ* may be a useful resource to develop as an agent for preventing and treating nicotine addiction.
